# Free Boundary Hamiltonian Stationary Lagrangian Discs in ${\mathbb {C}}^2$

**DOI:** 10.1007/s12220-025-01962-0

**Published:** 2025-04-04

**Authors:** Filippo Gaia

**Affiliations:** Department of Mathematics, ETH Zurich, Rämistrasse 101, 8092 Zurich, Switzerland

**Keywords:** Hamiltonian stationary Lagrangian surfaces, Free boundary problems, Minimal immersions, 53D12, 53C42, 53C24, 58E12

## Abstract

Let $\Omega \subset {\mathbb {C}}^2$ be a smooth domain. We establish conditions under which a weakly conformal, branched $\Omega$-free boundary Hamiltonian stationary Lagrangian immersion *u* of a disc in ${\mathbb {C}}^2$ is a $\Omega$-free boundary minimal immersion. We deduce that if $u$ is a weakly conformal, branched $B_1(0)$-free boundary Hamiltonian stationary Lagrangian immersion of a disc with Legendrian boundary, then $u(D^2)$ is a Lagrangian equatorial plane disc. Furthermore, we present examples of $\Omega$-free boundary Hamiltonian stationary discs, demonstrating the optimality of our assumptions.

## Introduction

In this note, we study Lagrangian surfaces in ${\mathbb {C}}^2$ that are stationary points of the area functional with respect to Hamiltonian variations preserving the boundary of the surface within the boundary of a smooth open domain $\Omega$ in ${\mathbb {C}}^2$. Our main result is the following.

### Theorem 1

Let $\Omega \subset {\mathbb {C}}^2$ be smooth domain. Let $u\in C^1\cap W^{2,1}(\overline{D}^2, {\mathbb {C}}^2)$ be a weakly conformal, Lagrangian immersion away from finitely many branch points in $D^2$, with continuous Lagrangian angle $\overline{g}$. Assume that *u* is $\Omega$-free boundary Hamiltonian stationary with the localization property. Moreover assume that

1 $$\begin{aligned} \langle \partial _\tau u, I(N\circ u)\rangle =0\text { on }\partial D^2, \end{aligned}$$

where *I* denotes the complex multiplication in ${\mathbb {C}}^2$ and *N* denotes the outer normal vector of $\Omega$. Then *u* is a calibrated, branched $\Omega$-free boundary minimal immersion.

For the definition of free boundary Hamiltonian stationary Lagrangian maps with the localization property we refer to Definition 1.

In the special case $\Omega =B_1(0)$ we deduce the following rigidity result.

### Corollary 2

Let $u\in C^1\cap W^{2,1}(\overline{D}^2, {\mathbb {C}}^2)$ be a weakly conformal, Lagrangian immersion away from finitely many branch points in $D^2$, with continuous Lagrangian angle $\overline{g}$. Assume that *u* is $B_1(0)$-free boundary Hamiltonian stationary with the localization property and that (1) is satisfied. Then $u(D^2)$ is a flat equatorial Lagrangian disc (i.e. it is the intersection of $B_1(0)$ with a Lagrangian 2-plane passing through the origin).

The two results are proved in Sect. 3.

Notice that if $\Omega =B_1(0)$, then condition (1) corresponds to requiring that the boundary curve $u\vert _{\partial D^2}$ is *Legendrian* with respect to the standard contact structure of ${\mathbb {S}}^3$.

Theorem 1 is optimal in the following sense: if $\overline{g}$ is not assumed to be continuous, there are examples of conformal, $B_1(0)$-free boundary Hamiltonian stationary Lagrangian maps in $C^1\cap W^{2,1}(\overline{D}^2, {\mathbb {C}}^2)$ with the localization property, which are immersions away from a single point but are not minimal. We will see in Sect. 4.1 that the Schoen–Wolfson cones constitute a family of such examples. Additionally, in Sect. 4.2 we will discuss examples of smooth domains $\Omega$ and of smooth, conformal $\Omega$-free boundary Hamiltonian stationary Lagrangian immersions with the localization property which do not satisfy (1) and are not minimal.

The proof of the two results is based on the observation that if *u* is a $\Omega$-free boundary Hamiltonian stationary Lagrangian immersion away from finitely many branch points and singular points, and satisfies (1), then

$$\begin{aligned} (N\circ u)\wedge \partial _\nu u=0\quad \text { and }\quad i\overline{g}\partial _\nu g=0\text { on }\partial D^2 \end{aligned}$$

(see Theorem 7).

The free boundary problem for smooth Lagrangian surfaces whose boundary lies either in a minimal Lagrangian submanifold or in a complex hypersurface in an 2*n*-dimensional Calabi–Yau manifold (for any *n*) has been studied by Schoen in [11]. In particular he showed that if $\Sigma$ is a free boundary Lagrangian stationary submanifold, then the conormal vector of $\Sigma$ along $\partial \Sigma$ is orthogonal to the constraint manifold, moreover in the first case its Lagrangian angle is constant on $\partial \Sigma$, while in the second case the normal derivative of the Lagrangian angle vanishes on $\partial \Sigma$. In both cases he deduced that $\Sigma$ is special Lagrangian and therefore free boundary minimal and calibrated.

A rigidity result analogous to Corollary 2 has been established by Nitsche in [8] (for $n=3$) and by Fraser and Schoen in [4] (for any *n* and in any space form):

### Theorem 3

(Theorem 1.1 in [4]) Let $u: D^2 \rightarrow B_1^n(0)$ be a proper branched minimal immersion such that $u(D^2)$ meets $\partial B_1^n(0)$ orthogonally. Then, $u(D^2)$ is an equatorial plane disk.

Similar rigidity results for capillary stable surfaces, without a priori assumptions on the topology of the surfaces, have been obtained by Ros and Souam in [10] (see also [14]). Corollary 2 can be deduced from Theorem 1 by means of Theorem 3; we will however provide a different proof, which is based on the fact that the Lagrangian angle of the immersion is constant.

More recently, Li, Wang and Weng showed in [6] that if $u: \overline{D^2}\rightarrow \overline{B_1^4(0)}$ is a branched, minimal, Lagrangian immersion with Legendrian capillary boundary on ${\mathbb {S}}^3$, then $u(\overline{D^2})$ is an equatorial plane disc. Their work was motivated by the study of Lagrangian surfaces in a symplectic manifold *M* with $\omega$-convex boundary (see Sect. 1.5 in [3] for the definition and some examples). In particular they suggested to look at free boundary (or capillary boundary) Lagrangian surfaces in such manifolds, focusing on the case of Lagrangian surfaces with Legendrian boundary in $B_1(0)$. Further rigidity results in this setting have been obtained by Luo and Sun in [7].

So far, the results available in the literature have concerned smooth free boundary Hamiltonian stationary Lagrangian surfaces. However, Schoen and Wolfson showed in [12] that even $W^{1,2}$ minimizers of the area among Lagrangian maps are smooth only away from a locally finite set of points, consisting of branch points and singular points having a Schoen–Wolfson cone as tangent map. Examples of $B_1(0)$-free boundary Hamiltonian stationary surfaces with singularities are given for instance by the Schoen–Wolfson cones (see Lemma 10).

We also recall that any weakly conformal, Hamiltonian stationary Lagrangian map in $W^{1,2}$ with isolated singularities lies locally in $C^{1,\sqrt{2}-1}$ (see Lemma V.3 in [5]). Therefore it seems natural to study the $\Omega$-free boundary Hamiltonian stationary problem in the class of $C^1$ Lagrangian maps which are immersions outside of isolated singular points and branch points in $D^2$. For our results we assumed *u* to be of class $C^1$ up to the boundary, in order to be able to exclude branch points or singularities on $\partial D^2$ and to make sense of the trace of *g* on $\partial D^2$. The regularity assumption $u\in W^{2,1}(D^2, {\mathbb {C}}^2)$ seems to be necessary to deduce the Euler–Lagrangian equation (10) from the condition that *u* is $\Omega$-free boundary Hamiltonian stationary with the localization property (see the proof of Lemma 5).

The localization property, introduced in Definition 1, appears to be a natural assumption for studying variations in the target by means of a mapping approach. This concept has been introduced by Rivière in [9] to study target harmonic maps.

We conclude the Introduction by listing some related open questions:

1. Is it possible to show the same results under weaker regularity assumptions on *u* and *g*? Ideally, one would like to assume only $u\in W^{1,2}$ and *g* continuous. Notice that in the classical (non-Lagrangian) case, $B_1(0)$-free boundary maps in $W^{1,2}$ (i.e. harmonic extensions of half-harmonic maps in $H^\frac{1}{2}(\partial D^2, {\mathbb {S}}^n)$) are smooth up to the boundary thanks to [2], thus they define possibly branched free boundary minimal immersions in the smooth sense. Then the rigidity result of Nitsche and Fraser–Schoen applies.
2. Are there examples of $B_1(0)$-free boundary Hamiltonian stationary Lagrangian surfaces with more than one singularity? In this regard, we remark that in [5] we constructed Hamiltonian stationary Lagrangian surfaces with multiple singularities, which, in case of real-analytic boundary data, are free boundary Hamiltonian stationary Lagrangian surfaces for a holomorphic curve as constraint manifold.
3. Can condition (1) in Corollary 2 be weakened or removed?

## Preliminaries

Denote by $z_1=x_1+iy_1$, $z_2=x_2+iy_2$ the two complex coordinates of ${\mathbb {C}}^2$. Let $\omega =dx_1\wedge dy_1+dx_2\wedge dy_2$ be the standard symplectic form on ${\mathbb {C}}^2$. A surface $\Sigma \subset {\mathbb {C}}^2$ is *Lagrangian* if $\omega \vert _\Sigma =0$. Analogously, a map $u\in W^{1,2}(D^2,{\mathbb {C}}^2)$ is *Lagrangian* if $u^*\omega =0$ a.e.

A map $u\in W^{1,1}(D^2,{\mathbb {C}}^2)$ is said to be *weakly conformal* if $\langle \partial _xu, \partial _yu\rangle =0$ and $|\partial _xu|^2=|\partial _yu|^2$ a.e..

If $u\in W^{1,2}(D^2,{\mathbb {C}}^2)$ is Lagrangian and weakly conformal, there exists a measurable function $g: D^2\rightarrow {\mathbb {S}}^1$ such that

2 $$\begin{aligned} u^*(dz_1\wedge dz_2)=e^{2\lambda }\overline{g}\, dx\wedge dy \text { a.e.}, \end{aligned}$$

where $e^{2\lambda }=|\partial _x u|^2=|\partial _y u|^2$ (see for instance p. 3 in [13]). The function $\overline{g}$ is called the *Lagrangian angle* of *u*. If a weakly conformal Lagrangian map *u* has constant Lagrangian angle, *u* is said to be *special Lagrangian*. If *u* is a special Lagrangian branched immersion, then *u* is a *calibrated* minimal branched immersion (see p. 4 in [13]). By a *minimal branched immersion* we mean here a non-constant smooth map $u\in C^\infty (D^2,{\mathbb {C}}^2)$ which is weakly conformal and harmonic, so that it parametrizes a smooth minimal surface outside of isolated points in $D^2$.

It will be convenient to identify ${\mathbb {C}}^2$ with the algebra of quaternions ${\mathbb {H}}$ with basis elements 1, *I*, *J*, *K*. In this identification, *I* corresponds to the complex multiplication. For any weakly conformal, Lagrangian $u\in W^{1,2}(D^2)$, (2) implies

3 $$\begin{aligned} \frac{1}{r}\partial _\theta u=-\overline{g}J\partial _r u\text { a.e.,} \end{aligned}$$

so that

4 $$\begin{aligned} \operatorname {div}({g}\nabla u)=0. \end{aligned}$$

Let $u\in C^1(\overline{D}^2,{\mathbb {C}}^2)$ be weakly conformal; we will say that *u* is *Lagrangian stationary* if for any $\tilde{u}\in C^1((-\varepsilon ,\varepsilon ),C^2(\overline{D}^2,{\mathbb {C}}^2))$ with

1. $$\tilde{u}(0,\cdot )=u$$
2. $\tilde{u}(t,\cdot )$ is Lagrangian for any *t*
3. $\tilde{u}(t,\cdot )=u$ outside of a compact set $K\subset D^2$ for any *t*

we have

5 $$\begin{aligned} \frac{d}{dt}\bigg \vert _{t=0}\frac{1}{2}\int _{D^2}|\nabla \tilde{u}(t,x)|^2 dx=0. \end{aligned}$$

If *u* is a conformal immersion, this corresponds to requiring that $u(D^2)$ is stationary for the area with respect to compactly supported Lagrangian variations.

Let $\Omega \subset {\mathbb {C}}^2$ be an open subset with smooth boundary. We will say that *u* is $\Omega$-* free boundary Lagrangian stationary* if (5) holds for any $\tilde{u}$ satisfying (1) and (2) above and such that $\frac{\partial }{\partial t}\big \vert _{t=0} \tilde{u}(t,x)$ is tangent to $\partial \Omega$ for any $x\in \partial D^2$.

We will also need the following definition:^1^

### Definition 1

A weakly conformal Lagrangian map $u\in C^1(\overline{D^2}, {\mathbb {C}}^2)$ with $u(\partial D^2)\subset \partial \Omega$ is $\Omega$-* free boundary Hamiltonian stationary with the localization property* if for every smooth, relatively open domain $\Lambda \subset \overline{D^2}$ such that $\partial \Lambda$ contains no singularities of *u*,

6 $$\begin{aligned} \int _\Lambda \langle d (I(\nabla f)\circ u); du\rangle =0 \end{aligned}$$

for any smooth function $f:{\mathbb {C}}^2\rightarrow {\mathbb {R}}$ supported away from $u(\partial \Lambda \cap D^2$) such that $\langle I\nabla f(x), N(x)\rangle =0$ for any *x* in a neighborhood of $u(\overline{\Lambda }\cap \partial D^2)$ in $\partial \Omega$, where *N* denotes the outer normal vector of $\Omega$. The notation $\langle \cdot ;\cdot \rangle$ is used to denote the scalar product on $T^*D^2\otimes {\mathbb {C}}^2$.

If (6) holds for any smooth $\Lambda \Subset D^2$, *u* is *Hamiltonian stationary with the localization property*.

Simple examples of $\Omega$-free boundary Hamiltonian stationary maps with the localization property can be constructed as follows. Let $D\subset {\mathbb {C}}$ be a smooth domain. Let $i: D\rightarrow {\mathbb {C}}^2$ be the embedding of *D* resulting from identifying ${\mathbb {C}}$ with ${\mathbb {R}}\times \{0\}\times {\mathbb {R}}\times \{0\}\subset {\mathbb {C}}^2$. Let $A\in U(2)$ and set $u:=A\circ i$. Let $\Omega \subset {\mathbb {C}}^2$ be a smooth domain such that $u(\partial D)\subset \partial \Omega$ and such that on $\partial D$ there holds $\partial _\nu u\wedge (N\circ u)=0$ (where $\nu$ denotes the outer normal vector of *D* and *N* the outer normal vector of $\Omega$). Then *u* is a conformal, $\Omega$-free boundary Hamiltonian stationary Lagrangian map with the localization property. Further examples are discussed in Section 4.

By the next Lemma, this property is satisfied by any $\Omega$-free boundary Lagrangian stationary map.

### Lemma 4

Let $u\in C^1(\overline{D}^2, {\mathbb {C}}^2)$ be a weakly conformal Lagrangian map. If *u* is ($\Omega$-free boundary) Lagrangian stationary, then *u* is ($\Omega$-free boundary) Hamiltonian stationary with the localization property.

### Proof

Assume that *u* is a weakly conformal ($\Omega$-free boundary) Lagrangian stationary map. Let $\Lambda \Subset D^2$ be a subdomain ($\Lambda \subset \overline{D^2}$ in the case of $\Omega$-free boundary maps) and let *f* be a smooth map on ${\mathbb {C}}^2$ supported away from $u(\partial \Lambda \cap D^2)$ (in the $\Omega$-free boundary case we also require that $\langle I\nabla f(x), N(x)\rangle =0$ for any *x* in a neighborhood of $u(\overline{\Lambda }\cap \partial D^2)$ in $\partial \Omega$). Let $\tilde{u}(t,x)$ be the solution of

7 $$\begin{aligned} {\left\{ \begin{array}{ll} \partial _t \tilde{u}(t,x)=I\nabla f(\tilde{u}(t,x))\\ \tilde{u}(0,x)=u(x) \end{array}\right. } \end{aligned}$$

for any $x\in \overline{\Lambda }$ and set $\tilde{u}(t,x)=u(x)$ for any $x\in \overline{D}^2\smallsetminus \Lambda$. We claim that $\tilde{u}$ is a variation of *u* through Lagrangian maps. In fact8as  and $d\omega =0$. Since $u^*\omega =0$ we conclude that $\tilde{u}(\cdot , t)^*\omega =0$ for any *t*. Moreover in the free boundary case $\frac{\partial }{\partial t}\big \vert _{t=0}\tilde{u}(t,x)=I\nabla f(u(x))\in T_{u(x)}\partial \Omega$ for any $x\in \partial D^2$. Thus $\tilde{u}$ is an admissible variation, and as *u* is ($\Omega$-free boundary) Lagrangian stationary, we have

9 $$\begin{aligned} \int _{\Lambda } \langle d(I(\nabla f)\circ u); du\rangle =\frac{d}{dt}\bigg \vert _{t=0}\frac{1}{2}\int _{D^2}|\nabla \tilde{u}(x,t)|^2\, dx=0. \end{aligned}$$

This shows that *u* is ($\Omega$-free boundary) Hamiltonian stationary with the localization property. $\square$

Notice that if $u\in C^1(\overline{D^2},{\mathbb {C}}^2)$ is a conformal immersion, then *g* is continuous in $\overline{D}^2$ since $\overline{g}=e^{-2\lambda }\det (\nabla u)$.

### Definition 2

Let $u\in C^1(\overline{D^2},{\mathbb {C}}^2)$ be a weakly conformal Lagrangian map such that $\nabla u$ vanishes at isolated points.

We say that $p\in \overline{D^2}$ is a *branch point* of *u* if $\nabla u(p)=0$ and the Lagrangian angle $\overline{g}$ of *u* is continuous at *p*.

We say that $p\in \overline{D^2}$ is a *singular point* of *u* if its Lagrangian angle $\overline{g}$ is not continuous at *p*.

In particular, if *p* is a singular point of *u*, then $\nabla u(p)=0$.

### Lemma 5

Let $u\in C^1\cap W^{2,1}(\overline{D}^2, {\mathbb {C}}^2)$ be a weakly conformal branched immersion away from

singular points $q_1,\ldots ,q_M\in D^2$ and branch points which do not accumulate at any point in $D^2$, with Lagrangian angle $\overline{g}$. Assume that *u* is Hamiltonian stationary with the localization property. Then $\overline{g}\in C^\infty _{\text {loc}}(D^2\smallsetminus \{q_1,\ldots ,q_M\})$ and satisfies

10 $$\begin{aligned} \operatorname {div}(\overline{g}\nabla g)=0\quad \text { and }\quad \operatorname {div}(i\overline{g}\nabla ^\perp g)=0\text { in }D^2\smallsetminus \{q_1,\ldots , q_M\}. \end{aligned}$$

If *u* has no singularities, there exists a harmonic function $\beta$ on $D^2$, continuous up to the boundary, such that $g=e^{i\beta }$.

If we also assume that *g* lies in $W^{1,1}(D^2)$, we have

11 $$\begin{aligned} \operatorname {div}(\overline{g}\nabla g)=\sum _{i=1}^M\alpha _i\delta _{q_i}\quad \text { and }\quad \operatorname {div}(i\overline{g}\nabla ^\perp g)=2\pi \sum _{i=1}^Md_i\delta _{q_i}\text { in }D^2 \end{aligned}$$

for $\alpha _1,\ldots ,\alpha _M\in {\mathbb {R}}$ and integers $d_1,\ldots ,d_M$.

### Proof

Assume first that *u* has no singularities. Let $\{p_i\}_{i\in I}$ denote the countably many branch points of *u* in $D^2$ and let $\Xi :=D^2\smallsetminus \bigcup _{i\in I}\{p_i\}$. Notice that since $u\in C^1(\overline{D^2},{\mathbb {C}}^2)$, *g* is continuous on $\Xi$. Since $\nabla u\in W^{1,1}(D^2)$, and $|\nabla u|$ is bounded from above and locally bounded away from zero in $\Xi$, $\overline{g}=\det \left( \frac{\nabla u}{|\nabla u|}\right) \in W^{1,1}_{\text {loc}}(\Xi )$. Let $\Lambda \Subset \Xi$ be a smooth subdomain and let *f* be a smooth function on ${\mathbb {C}}^2$ supported away from $u(\partial \Lambda )$. If *u* is Hamiltonian stationary with the localization property, then we have

12 $$\begin{aligned} 0=&\int _{\Lambda }\langle du; d(I(\nabla f)\circ u)\rangle =-\int _{\Lambda }\langle i\overline{g}dg\cdot du, (\nabla f)\circ u\rangle =-\int _{\Lambda } i\overline{g}dg\cdot d(f\circ u), \end{aligned}$$

where the second equality follows from (4). For any $p\in \Xi$ let $\Lambda _p$ be a subdomain as above containing *p* and such that *u* restricted to $\Lambda _p$ is a $C^1$ diffeomorphism to its image. Notice that any $\varphi \in C_c^1(\Lambda _p)$ can be written as $\varphi =f_\varphi \circ u$ for some $C^1$ function $f_\varphi$ on ${\mathbb {C}}^2$ supported away from $u(\partial \Lambda _p)$. Approximating $f_\varphi$ in $C^1$ by smooth functions with the same properties we obtain

13 $$\begin{aligned} -\int _\Lambda i\overline{g}dg\cdot d\varphi =-\int _\Lambda i\overline{g}dg\cdot d(f_\varphi \circ u)=0. \end{aligned}$$

Therefore

14 $$\begin{aligned} \operatorname {div}(\overline{g}\nabla g)=0 \end{aligned}$$

on $\Lambda _p$, and since the argument holds for any $p\in \Xi$ we conclude that (14) holds in $\Xi$. Since *g* is continuous, there exists $\beta \in C^0(\overline{D^2})$ (unique up to addition of a constant in $2\pi {\mathbb {Z}}$) such that $g=e^{i\beta }$. By (14), $\beta$ satisfies $\Delta \beta =0$ in $\Xi$. Since $\beta$ is bounded and has isolated singularities, $\beta$ is harmonic in $D^2$. In particular, we have

15 $$\begin{aligned} \operatorname {div}(i\overline{g}\nabla ^\perp g)=-\operatorname {div}(\nabla ^\perp \beta )=0\text { in }D^2. \end{aligned}$$

Next assume that *u* is an immersion away from singular points $q_1,\ldots ,q_M\in D^2$ and branch points which do not accumulate at any point in $D^2$. Arguing as in the first part of the proof we see that on any simply connected, smooth, open $\Lambda \subset \Psi :=D^2\smallsetminus \{q_1,\ldots ,q_M\}$ there exists a harmonic function $\beta$ such that $g=e^{i\beta }$ on $\Lambda$, so that $\operatorname {div}(\overline{g}\nabla g)=0$ and $\operatorname {div}(\overline{g}\nabla ^\perp g)=0$ on $\Psi$. Arguing as in Lemma 2 in [1] we obtain that

16 $$\begin{aligned} \operatorname {div}(ig\nabla \overline{g})=\sum _{i=1}^M\alpha _i\delta _{q_i}\text { and }\operatorname {div}(i\overline{g}\nabla ^\perp g)=2\pi \sum _{i=1}^Md_i\delta _{q_i}\text { in }D^2 \end{aligned}$$

for $\alpha _1,\ldots ,\alpha _M\in {\mathbb {R}}$ and integers $d_1,\ldots ,d_M$ (these are the degrees of the singular points, see [1] for details). $\square$

### Remark 6

Let $u\in C^1\cap W^{2,1}(\overline{D^2},{\mathbb {C}}^2)$ be a weakly conformal, Lagrangian immersion away from finitely many singular points and branch points in $D^2$, with Lagrangian angle $\overline{g}$. Assume that *u* is Hamiltonian stationary with the localization property. Let $\Lambda \subset \overline{D^2}$ be a relatively open, simply connected, smooth subset such that $\overline{\Lambda }$ contains no singularities of *u*. Then $\overline{g}$ is continuous on $\overline{\Lambda }$ and it can be written as $\overline{g}=e^{-i\beta }$ for a harmonic function $\beta \in W^{1,1}(\Lambda )\cap C^1(\overline{\Lambda })$. Therefore there exists $G_\Lambda \in W^{1,1}(\Lambda )$ such that $-i\overline{g}\nabla g=\nabla \beta =\nabla ^\perp G_\Lambda$ in $\Lambda$ (i.e. $G_\Lambda$ is the harmonic conjugate of $\beta$ in $\Lambda$).

Notice that since $\beta$ is continuous on $\partial \Lambda$, for any $p\in (1,\infty )$ there holds $G_\Lambda \in L^p(\partial \Lambda )$ with $\Vert G_\Lambda \Vert _{L^p(\partial \Lambda )}\le C(p)\Vert \beta \Vert _{L^p(\partial \Lambda )}$ (by continuity of the Hilbert transform in $L^p(\partial \Lambda )$).

For any $\varphi \in C^1(\overline{\Lambda })$ there holds

17 $$\begin{aligned} \int _{\Lambda }i\overline{g}\nabla g\cdot \nabla \varphi =-\int _\Lambda \nabla ^\perp G_\Lambda \cdot \nabla \varphi =\int _{\partial \Lambda }G_\Lambda \partial _\tau \varphi , \end{aligned}$$

where $\tau$ denote the oriented unit tangent vector on $\partial \Lambda$.

Let $u\in C^1\cap W^{2,1}(\overline{D^2},{\mathbb {C}}^2)$ be a weakly conformal, Lagrangian immersion away from finitely many singular points and branch points in $D^2$, with Lagrangian angle $\overline{g}$. Assume that *u* is Hamiltonian stationary with the localization property. Let $\{\Lambda _i\}_{i=1}^Q$ be a covering of $\partial D^2$ by relatively open, simply connected, smooth subset of $\overline{D^2}$ containing no singular point of *u*, as in Remark 6, and let $\Lambda _0\Subset D^2$ such that $\overline{D^2}=\bigcup _{i=0}^Q\Lambda _i$. Let $\{\psi _i\}_{i=0}^Q$ be a partition of unity subordinate to $\{\Lambda _i\}_{i=0}^Q$. For any $\varphi \in C^1(\partial D^2,{\mathbb {C}}^2)$ we define

18 $$\begin{aligned} \langle i\overline{g}\partial _\nu g, \varphi \rangle :=\sum _{i=1}^Q\int _{\Lambda _i}i\overline{g}\nabla g\cdot \nabla (\tilde{\varphi }\psi _i) =\sum _{i=1}^Q\int _{\partial \Lambda _i}G_{\Lambda _i}\partial _\tau (\varphi \psi _i), \end{aligned}$$

where $\tilde{\varphi }$ is any $C^1$ extension of $\varphi$ in $D^2$ supported away form the singularities of *u*. The following estimate holds:

19 $$\begin{aligned} |\langle i\overline{g}\partial _\nu g, \varphi \rangle |\le C\sum _{i=1}^Q \Vert G_{\Lambda _i}\Vert _{L^1(\partial \Lambda _i\cap \partial D^2)}\Vert \varphi \Vert _{C^1(\partial D^2)}, \end{aligned}$$

therefore $i\overline{g}\partial _\nu g$ defines a distribution on $\partial D^2$. If $\overline{g}\in C^1(\overline{D^2})$, then $\langle i\overline{g}\partial _\nu g, \varphi \rangle =\int _{\partial D^2}i\overline{g}\partial _\nu g\varphi$. The two expressions in (18) show that the definition of $i\overline{g}\partial _\nu g$ does not depend on the choice of the sets $\Lambda _i$, on the partition if unity or on the extension $\tilde{\varphi }$ of $\varphi$.

Notice that if for any $i\in \{1,\ldots ,Q\}$ $G_{\Lambda _i}$ is constant on $\overline{\Lambda _i}\cap \partial D^2$, then integration by parts implies that $i\overline{g}\partial _\nu g=0$.

## The Main Results

Theorem 1 and Corollary 2 will be deduced from the following characterization result.

### Theorem 7

Let $\Omega \subset {\mathbb {C}}^2$ be a smooth domain. Let $u\in C^1\cap W^{2,1}(\overline{D}^2, {\mathbb {C}}^2)$ be a weakly conformal, Lagrangian immersion away from finitely many singular points and finitely many branch points in $D^2$, with Lagrangian angle $\overline{g}$. Assume that *u* is $\Omega$-free boundary Hamiltonian stationary with the localization property. Assume in addition that

20 $$\begin{aligned} \langle \partial _\tau u, I(N\circ u)\rangle =0\text { on }\partial D^2. \end{aligned}$$

Then

21 $$\begin{aligned} (N\circ u)\wedge \partial _\nu u=0\text { on }\partial D^2 \end{aligned}$$

and

22 $$\begin{aligned} i\overline{g} \partial _\nu g =0\text { on }\partial D^2, \end{aligned}$$

where $i\overline{g}\partial _\nu g$ is defined as in (18).

Conversely, if $\overline{g}$ lies in $W^{1,1}(D^2)$ and satisfies

23 $$\begin{aligned} \operatorname {div}(i\overline{g}\nabla g)=0 \end{aligned}$$

as well as equations (21) and (22), then *u* is $\Omega$-free boundary Hamiltonian stationary with the localization property.

### Proof

Assume first that *u* is $\Omega$-free boundary Hamiltonian stationary Lagrangian with the localization property and that (20) holds.

Let $\Lambda \subset \overline{D^2}$ be a relatively open, simply connected, smooth domain such that $\overline{\Lambda }$ does not contain any singular

point of *u*. Then *g* is continuous on $\overline{\Lambda }$. Let *f* be a smooth function on ${\mathbb {C}}^2$ supported away from $u(\partial \Lambda \cap D^2)$ and such that $\langle I\nabla f(x), N(x)\rangle =0$ for any *x* in a neighborhood of $u(\overline{\Lambda }\cap \partial D^2)$. Then we have

24 $$\begin{aligned} 0=&\int _{\Lambda }\langle du; d(I(\nabla f)\circ u)\rangle =\int _{\overline{\Lambda }\cap \partial D^2}\langle \partial _\nu u, I(\nabla f)\circ u\rangle +\int _{\Lambda }\langle \overline{g}dg\cdot d u, I(\nabla f)\circ u\rangle \\ \nonumber =&\int _{\overline{\Lambda }\cap \partial D^2}\langle \partial _\nu u, I(\nabla f)\circ u\rangle -\int _\Lambda i\overline{g}dg\cdot d(f\circ u)\\ \nonumber =&\int _{\overline{\Lambda }\cap \partial D^2}\langle \partial _\nu u, I(\nabla f)\circ u\rangle -\int _{\partial \Lambda }G_\Lambda \partial _\tau (f\circ u), \end{aligned}$$

where the second equality follows from (4)^2^ and the function $G_\Lambda$ in the last expression is the one introduced in Remark 6. Notice that by the assumptions on *f*, in the second term of the right hand side the integrand vanishes on $\partial \Lambda \cap D^2$. Thus for $\sigma :=\overline{\Lambda }\cap \partial D^2$,

25 $$\begin{aligned} \int _{\sigma } \langle \partial _{\nu }u, I(\nabla f)\circ u\rangle =\int _\sigma G_\Lambda \partial _\tau (f\circ u) \end{aligned}$$

for any smooth *f* supported away from $u(\partial \sigma )$ and such that $\langle I\nabla f(x), N(x)\rangle =0$ for any *x* in a neighborhood of $u(\sigma )$. In particular, (25) holds for any segment $\sigma \subset \partial D^2$ (for *f* as above).

We claim that on $\partial D^2$ there holds $\langle \partial _\nu u, I(N\circ u)\rangle =0$. To see this, let

26 $$\begin{aligned} \Sigma _+:=\left\{ x\in \partial D^2\vert \langle \partial _\nu u(x), IN(u(x))\rangle >0\right\} . \end{aligned}$$

Notice that $\Sigma _+$ is an open subset of $\partial D^2$. Moreover, for any $n\in {\mathbb {N}}$ set

27 $$\begin{aligned} \Sigma _+^\frac{1}{n}:=\left\{ x\in \Sigma _+\vert \operatorname {dist}(x,\partial \Sigma _+)>\frac{1}{n}\right\} . \end{aligned}$$

For any $n\in {\mathbb {N}}$ let $\varphi _n$ be a smooth non-negative function supported on $\Sigma _+$ and such that $\varphi _n\equiv 1$ on $\Sigma _+^\frac{1}{n}$ and let $f_n$ be a smooth function defined on ${\mathbb {C}}^2$, supported away from $u(\partial \Lambda \cap D^2)$ and such that

28 $$\begin{aligned} {\left\{ \begin{array}{ll} f_n\equiv 0& \text { on }\partial \Omega \\ \nabla f_n(x)=\varphi _n(x)N(x)& \text { on }\partial \Omega . \end{array}\right. } \end{aligned}$$

Then (25) implies that for any segment $\sigma$ as above

29 $$\begin{aligned} \int _{\sigma \cap \Sigma _+^\frac{1}{n}}|\langle \partial _\nu u, I(N\circ u)\rangle |\le \int _{\sigma \cap \Sigma _+} \langle \partial _{\nu }u, I(\nabla f_n)\circ u\rangle =0. \end{aligned}$$

As this holds for any $n\in {\mathbb {N}}$ and $\Sigma _+=\bigcup _{n\in {\mathbb {N}}}\Sigma _+^\frac{1}{n}$, we conclude that $\Sigma _+$ has $\mathscr {H}^1$ measure zero. Similarly, one can show that the set of points *x* in $\partial D^2$ where $\langle \partial _\nu u(x), IN(u(x))\rangle <0$ also has measure zero. This proves the claim.

Notice that by assumption (20) and the fact that $\partial _{\tau }u(x)$ is a non-zero element of $T_{u(x)}\partial \Omega$ for any $x\in \partial D^2$, we have that

$$\begin{aligned} \left\{ N(u(x)), IN(u(x)), \partial _\tau u(x), I\partial _\tau u(x)\right\} \end{aligned}$$

is an orthogonal basis of $T_{u(x)}{\mathbb {C}}^2$ for any $x\in \partial D^2$. For any $x\in \partial D^2$, $\langle IN(u(x)),\partial _\nu u(x)\rangle =0$ by the previous claim, $\langle I\partial _\tau u(x),\partial _\nu u(x)\rangle =0$ as *u* is Lagrangian and $\langle \partial _\tau u(x), \partial _\nu u(x)\rangle =0$ as *u* is conformal. We conclude that $\partial _\tau u(x)\wedge N(u(x))=0$, this shows (21).

Next we claim that $i\overline{g}\partial _{\nu }g=0$ on $\partial D^2$ in the sense of (18). We will show that for any $x\in \partial D^2$, there exists $\Lambda$ as above such that $\overline{\Lambda }$ contains a neighborhood of *x* in $\partial D^2$ and $G_\Lambda$ is constant on $\overline{\Lambda }\cap \partial D^2$.

Assume first that *u* is smooth on $\overline{D^2}$, up to the boundary. Notice that on $\partial D^2$

$$\begin{aligned} \langle \partial _\tau u, I(N\circ u)\rangle =-\langle \overline{g}J\partial _\nu u, I(N\circ u)\rangle =0 \end{aligned}$$

by (3) and (21). For any $x\in \partial D^2$, let $\Lambda$ be a domain as above with $x\in \Lambda$, such that $\overline{\Lambda }\cap \partial D^2$ is connected and such that *u* is injective on $\overline{\Lambda }\cap \partial D^2$. Let $\alpha$ be a smooth function on $\overline{\Lambda }\cap \partial D^2$ supported away from $\partial (\overline{\Lambda }\cap \partial D^2)$ and such that $\int _{\overline{\Lambda }\cap \partial D^2}\alpha =0$. Then there exists a smooth function *f* on ${\mathbb {C}}^2$ such that $\langle \nabla f, IN\rangle =0$ in a neighborhood of $u(\overline{\Lambda }\cap \partial D^2)$ in $\partial \Omega$ and $\partial _\tau (f\circ u)=\alpha$ on $\overline{\Lambda }\cap \partial D^2$. Plugging *f* into (25) and varying $\alpha$ we obtain that $G_\Lambda$ is constant on $\overline{\Lambda }\cap \partial D^2$. This argument, however, fails when *u* is only of class $C^1\cap W^{2,1}(\overline{D}^2, {\mathbb {C}}^2)$.

For the general case we proceed as follows. Given $x\in \partial D^2$ set $p:=u(x)$, $v=\frac{\partial _\tau u(x)}{|\partial _\tau u(x)|}$. Let $g_0\in {\mathbb {S}}^1\subset {\mathbb {C}}$ such that $v=g_0JN(p)$ (recall that $\langle \partial _\tau u(x), N(u(x))\rangle =0$, $\langle \partial _\tau u(x), IN(u(x))\rangle =0$ on $\partial D^2$). Let *P* be the hyperplane passing through *p* and orthogonal to *v*. Extend *N* to a smooth vector field in a neighborhood of *p* in ${\mathbb {C}}^2$ (still denoted by *N*). Notice that for $\varepsilon >0$ sufficiently small, the flow of $g_0JN$ induces a diffeomorphism $\Psi$ from $B^P_\varepsilon (p)\times (-\delta , \delta )$ to a neighborhood *U* of *p* (where $B^P_\varepsilon (p)$ denotes a ball in the plane *P*). If $\delta$ is chosen sufficiently small, $\Psi$ satisfies

1. $\partial _t\Psi =g_0J(N\circ \Psi )$ (*t* denotes the coordinate in $(-\delta , \delta )$);
2. on the connected component $\sigma$ of $u^{-1}(U)\cap \partial D^2$ containing *x*, there holds
  $$\begin{aligned} \left\langle \frac{\partial _\tau u(y)}{|\partial _\tau u(y)|}, g_0J(N\circ u)(y)\right\rangle >\frac{1}{2}. \end{aligned}$$

Let $\Lambda$ be a smooth, simply connected, relatively open subset of $\overline{D^2}$ which contains no singular or branch point of *u* and such that $x\in \Lambda$ and $\overline{\Lambda }\cap \partial D^2=\sigma$. For any $\alpha \in C^\infty _c((-\delta , \delta ))$ with $\int _{-\delta }^\delta \alpha =0$ let $\beta \in C^\infty _c((-\delta , \delta ))$ such that $\beta '=\alpha$. Set

$$\begin{aligned} \tilde{\beta }: B^P_\varepsilon (p)\times (-\delta , \delta )\rightarrow {\mathbb {R}}, \quad (x,t)\mapsto \beta (t). \end{aligned}$$

Notice that on *U*

30 $$\begin{aligned} \nabla (\tilde{\beta }\circ \Psi ^{-1})(y) \wedge g_0JN(y)=0. \end{aligned}$$

Now let $\eta$ be a smooth cut-off function supported in *U* and such that $\eta \equiv 1$ in a neighborhood of

$$\begin{aligned} \overline{\{x\in u(\sigma )\vert \tilde{\beta }\circ \Psi ^{-1}(x)\ne 0\}}. \end{aligned}$$

Since by (21) $\partial _\nu u\wedge (N\circ u)=0$ on $\partial D^2$, (30) implies that

31 $$\begin{aligned} \langle N,I\nabla (\eta (\tilde{\beta } \circ \Psi ^{-1}))\rangle =0 \end{aligned}$$

in a neighborhood of $u(\sigma )$.

Notice also that $\eta (\tilde{\beta } \circ \Psi ^{-1})$ is a smooth, compactly supported function on ${\mathbb {C}}^2$, supported away from $u(\partial \sigma )$. Thus by (25) and (31)

32 $$\begin{aligned} \int _{\partial D^2}G_\Lambda \partial _\tau (\tilde{\beta }\circ \Psi ^{-1}\circ u) =\int _{\sigma }G_\Lambda \partial _\tau ((\eta (\tilde{\beta }\circ \Psi ^{-1}))\circ u) =0. \end{aligned}$$

We compute

$$\begin{aligned} \partial _\tau (\tilde{\beta }\circ \Psi ^{-1}\circ u)=\partial _t\tilde{\beta }(\Psi ^{-1}\circ u)\left\langle \left( \nabla \Psi ^{-1}\right) \circ u\cdot \partial _\tau u, \frac{\partial }{\partial t}\right\rangle , \end{aligned}$$

where $\cdot$ denotes the scalar product in the tangent bundle of *U*, while $\langle \cdot ,\cdot \rangle$ denotes the scalar product in the tangent bundle of $B_\varepsilon ^P(p)\times (-\delta , \delta )$.

Notice that the map

33 $$\begin{aligned} \pi : B^P_\varepsilon (p)\times (-\delta , \delta )\rightarrow \{p\}\times (-\delta , \delta ),\quad (x,t)\mapsto (p,t) \end{aligned}$$

is bi-Lipschitz when restricted to $(\Psi ^{-1}\circ u)(\sigma )$ by property (2) of $\Psi$, and

34 $$\begin{aligned} J\left( \pi \circ \Psi ^{-1}\circ u\vert _{\sigma }\right) =\left|\left\langle \left( \nabla \Psi ^{-1}\right) \circ u\cdot \partial _\tau u, \frac{\partial }{\partial t}\right\rangle \right|. \end{aligned}$$

Therefore, by means of a change of variables, we can rewrite (32) as

35 $$\begin{aligned}&\int _{-\delta }^\delta \alpha (t)\left( G_\Lambda \circ u\vert _\sigma ^{-1}\circ \Psi \circ (\pi \vert _{(\Psi ^{-1}\circ u)(\sigma )})^{-1}\right) (t)\, dt\\ \nonumber&\quad =\int _{-\delta }^\delta \partial _t\tilde{\beta }((\pi \vert _{(\Psi ^{-1}\circ u)(\sigma )})^{-1}(t))\left( G_\Lambda \circ u\vert _\sigma ^{-1}\circ \Psi \circ \left( \pi \vert _{(\Psi ^{-1}\circ u)(\sigma )}\right) ^{-1}\right) (t)\, dt =0. \end{aligned}$$

As (35) holds for any smooth function $\alpha$ with zero average, we conclude that $G_\Lambda$ is constant on $\overline{\Lambda }\cap \partial D^2$. As observed at the end of Sect. 2, this implies $i\overline{g}\partial _\nu g=0$.

On the other hand, assume that *g* lies in $W^{1,1}(D^2)$ and satisfies $\operatorname {div}(i\overline{g}\nabla g)=0$ in $D^2$. Let $\Lambda \subset \overline{D^2}$ be a smooth, relatively open subset such that $\partial \Lambda$ contains no singularities of *u* and let *f* be a smooth function on ${\mathbb {C}}^2$ supported away from $u(\partial \Lambda \cap D^2)$ and such that $\langle I\nabla f(x), N(x)\rangle =0$ for any *x* in a neighborhood of $u(\overline{\Lambda }\cap \partial D^2)$ in $\partial \Omega$. By computation (24) we have^3^

36 $$\begin{aligned} \int _{\Lambda }\langle du; d(I(\nabla f)\circ u)\rangle =\int _{\overline{\Lambda }\cap \partial D^2}\langle \partial _\nu u, I(\nabla f)\circ u\rangle -\langle i\overline{g}\partial _\nu g,f\circ u\vert _{\partial D^2}\rangle . \end{aligned}$$

Note that by the assumptions on *f*, in the second term of the right hand side the integrand vanishes on $\partial \Lambda \cap D^2$. We claim that if *u* satisfies (21) and (22), the right hand side in (36) vanishes. For the first term, notice that by the assumptions on *f* and by (21), $\langle I\nabla f(u(x)), N(u(x))\rangle =0$ and $N(u(x))\wedge \partial _\nu u(x)$ for any $x\in \overline{\Lambda }\cap \partial D^2$. Assumption (22) implies that the second term vanishes as well. We conclude that *u* is $\Omega$-free boundary Hamiltonian stationary with the localization property. $\square$

Theorem 1 is a consequence of Theorem 7:

### Corollary 8

Let $u\in C^1\cap W^{2,1}(\overline{D}^2, {\mathbb {C}}^2)$ be a weakly conformal, Lagrangian immersion away from finitely many branch points in $D^2$, with continuous Lagrangian angle $\overline{g}$. Then $\overline{g}$ is constant and *u* is a calibrated, branched $\Omega$-free boundary minimal immersion.

### Proof

By Lemma 5 the Lagrangian angle $\overline{g}$ of *u* can be written as $\overline{g}=e^{-i\beta }$ for some harmonic function $\beta$ on $D^2$. By (22) in Theorem 7, $i\overline{g}\partial _\nu g=0$ on $\partial D^2$ in the sense of (18). Therefore there exists a harmonic function $G_{D^2}$ such that $\nabla \beta =\nabla ^\perp G_{D^2}$ in $D^2$ and such that $G_{D^2}$ is constant on $\partial D^2$. This implies that $G_{D^2}$ is constant on $D^2$, therefore $\beta$ is also constant on $D^2$, say equal to $\beta _0\in {\mathbb {R}}$. Then *u* is special Lagrangian. As *u* is weakly conformal and satisfies $\Delta u=0$ (by (4)), it is a branched minimal immersion. Moreover, by Theorem 7 it satisfies $\partial _\nu u\wedge (N\circ u)=0$ on $\partial D^2$, therefore $u(\overline{D^2})$ is orthogonal to $\partial \Omega$ along $u(\partial D^2)$. We conclude that *u* is a calibrated, branched $\Omega$-free boundary minimal immersion. $\square$

For the special case $\Omega =B_1(0)$ we obtain Corollary 2:

### Corollary 9

Let $u\in C^1\cap W^{2,1}(\overline{D^2},{\mathbb {C}}^2)$ be a $B_1(0)$-free boundary Hamiltonian stationary Lagrangian immersion away from finitely many branch points in $D^2$, with the localization property. Assume that the boundary curve satisfies $\langle \partial _\tau u, Iu\rangle =0$ (i.e. the boundary curve is Legendrian). Then $u(\overline{D^2})$ is a Lagrangian flat equatorial disc (i.e. it is the intersection of $\overline{B_1(0)}$ with a Lagrangian 2-plane passing through the origin).

### Proof

Corollary 8 implies that *u* has constant Lagrangian angle, say equal to $e^{-i\beta _0}$ for some $\beta _0\in {\mathbb {R}}$, and is a weakly conformal branched $B_1(0)$-free boundary minimal immersion.

Notice that by (3) and (21)

$$\begin{aligned} \partial _\tau u=\pm |\partial _\tau u|e^{-i\beta _0}Ju\text { on }\partial D^2. \end{aligned}$$

Let $\gamma : [0,L)\rightarrow {\mathbb {S}}^3$ be an arclength parametrization of the curve $u\vert _{\partial D^2}$, then $\gamma$ satisfies $\dot{\gamma }=\pm e^{-i\beta _0}J\gamma$, and thus parametrizes a great circle in ${\mathbb {S}}^3$, i.e. the intersection of ${\mathbb {S}}^3$ with a 2-plane *P* passing through the origin. Since *u* satisfies (4) with constant Lagrangian angle, *u* is harmonic; thus by the maximum principle it takes values in the plane *P*, and therefore it is holomorphic (for the complex structure induced by an appropriate isometric identification of *P* with ${\mathbb {C}}$). By the maximum principle for holomorphic functions, *u* must take values in the disc $\overline{D}_\gamma$ spanned by $\gamma$. As its image is connected, we conclude that $u(\overline{D^2})$ is indeed the flat equatorial disc $\overline{D}_\gamma$.

Alternatively, once we know that *u* is a branched minimal immersion and satisfies $\partial _\nu u\wedge u=0$ on $\partial D^2$, the result follows from Theorem 2.1 in [4] or Theorem 1.1 in [6]. $\square$

## Examples of Free Boundary Hamiltonian Stationary Lagrangian Discs

### Schoen–Wolfson Cones

In this section we show that Schoen–Wolfson cones are $B_1(0)$-free boundary Hamiltonian stationary Lagrangian surfaces. This implies that the rigidity result of Corollary 2 doesn’t hold if we do not assume that the Lagrangian angle $\overline{g}$ is continuous.

Recall that for any relatively prime positive integers *p* and *q*, the Schoen–Wolfson cone $\Sigma _{p,q}$—introduced in [12]—has the following weakly conformal parametrization:

37 $$\begin{aligned} \Phi _{p,q}:\overline{D^2}\rightarrow {\mathbb {C}}^2,\quad r e^{i\theta }\mapsto \frac{r^{\sqrt{pq}}}{\sqrt{p+q}} \begin{pmatrix}\sqrt{q}e^{ip\theta } \\ i\sqrt{p}e^{-iq\theta }\end{pmatrix}. \end{aligned}$$

The derivatives of $\Phi _{p,q}$ are given by

38 $$\begin{aligned}&\partial _r\Phi _{p,q}(r,\theta )=\frac{\sqrt{pq} \,r^{\sqrt{pq}-1}}{\sqrt{p+q}} \begin{pmatrix}\sqrt{q}e^{ip\theta } \\ i\sqrt{p}e^{-iq\theta }\end{pmatrix}, \nonumber \\&\partial _\theta \Phi _{p,q}(r,\theta )=\frac{\sqrt{pq}\,r^{\sqrt{pq}}}{\sqrt{p+q}} \begin{pmatrix} i\sqrt{p}e^{ip\theta } \\ \sqrt{q}e^{-iq\theta } \end{pmatrix}, \end{aligned}$$

thus $\Phi _{p,q}$ is a Lagrangian map and its Lagrangian angle is given by

39 $$\begin{aligned} \frac{dz_1\wedge dz_2(\partial _r\Phi _{p,q}, \frac{1}{r}\partial _\theta \Phi _{p,q})}{|\partial _r\Phi _{p,q}\wedge \frac{1}{r}\partial _\theta \Phi _{p,q}|}=e^{i(p-q)\theta }. \end{aligned}$$

#### Lemma 10

For any relatively prime positive integers *p*, *q*, the map $\Phi _{p,q}$ is $B_1(0)$-free boundary Hamiltonian stationary Lagrangian with the localization property.

#### Proof

First we notice that the Lagrangian angle $\overline{g}=e^{i(p-q)\theta }$ is ${\mathbb {S}}^1$-harmonic: observe that $i\overline{g}\nabla g=-\nabla ^\perp \log (r^{p-q})$ distributionally, therefore

$$\begin{aligned} \operatorname {div}(i\overline{g}\nabla g)=-\operatorname {div}(\nabla ^\perp \log (r^{p-q})) =0. \end{aligned}$$

We also notice that $\Phi _{p,q}$ lies in $C^1\cap W^{2,1}(\overline{D^2})$, $\overline{g}$ lies in $W^{1,(2,\infty )}$ and is smooth outside of the origin, and $\Phi _{p,q}(\partial D^2)\subset {\mathbb {S}}^3$. Therefore, in view of Theorem 7, in order to show that $\Phi _{p,q}$ is a $B_1(0)$-free boundary Hamiltonian stationary Lagrangian map with the localization property, it is enough to check conditions (21) and and (22), i.e. that $i\overline{g}\partial _\nu g=0$ and $\partial _\nu \Phi _{p,q}\wedge \Phi _{p,q}=0$ on $\partial D^2$. The conditions can be verified directly from the explicit expressions (37), (38) and (39). $\square$

### Free Boundary Hamiltonian Stationary Lagrangian Surfaces with Non-constant Angle

The next result shows that if we remove the assumption that *u* satisfies $\partial _\tau u\cdot I(N\circ u)=0$ on $\partial D^2$ from Theorem 1, then the Theorem might fail. The construction of the following example is based on observations made in [5].

#### Lemma 11

There exist smooth, conformal, Lagrangian maps with continuous Lagrangian angle which are $\Omega$-free boundary Hamiltonian stationary with the localization property for some smooth domain $\Omega \subset {\mathbb {C}}^2$, but are not minimal.

#### Proof

Let $\varphi : \overline{D^2}\rightarrow {\mathbb {C}}$ be defined by

$$\begin{aligned} \varphi (x,y):=x \end{aligned}$$

and let $g:=e^{i\varphi }$. Then

$$\begin{aligned} \operatorname {div}(i\overline{g}\nabla g)=0, \end{aligned}$$

i.e. *g* is ${\mathbb {S}}^1$-harmonic. Let

$$\begin{aligned} G(x,y):= y \end{aligned}$$

and notice that

$$\begin{aligned} \nabla ^\perp G=i\overline{g}\nabla g. \end{aligned}$$

Set

$$\begin{aligned} u=\begin{pmatrix} \overline{g}\\ iG \end{pmatrix}. \end{aligned}$$

Then *u* is conformal and $\operatorname {div}(g\nabla u)=0$, i.e. *u* is a smooth conformal Hamiltonian stationary Lagrangian map with Lagrangian angle $\overline{g}$.

Observe that *u* is a smooth embedding of a disc, and $u\vert _{\partial D^2}$ defines a smooth embedded curve, which will be denoted by $\Gamma$. Consider the vector field

$$\begin{aligned} X:=\overline{g}J\partial _\tau u+G I\partial _\tau u \end{aligned}$$

defined along $\Gamma$. Notice that *X* doesn’t vanish and $\partial _\tau u\cdot X=0$ on $\Gamma$. Thus *X* is a non-vanishing section of the normal bundle of the smooth curve $\Gamma$. By the Tubular Neighborhood Theorem, we can identify a neighborhood of ${\mathbb {C}}^2$ around $\Gamma$ with a neighborhood of the zero section of the normal bundle $N\Gamma$ of $\Gamma$. Under this identification, we can define $\Omega$ as

$$\begin{aligned} \Omega :=\bigcup _{x\in \Gamma }B^{N_x\Gamma }_\varepsilon \left( x+\varepsilon \frac{X}{|X|}\right) , \end{aligned}$$

where $B^{N_x\Gamma }_\varepsilon (y)$ denotes an open ball in the fiber $N_x\Gamma$ of the normal bundle $N\Gamma$ at $x\in \Gamma$. Then $\Omega$ is a smooth domain in ${\mathbb {C}}^2$, $\Gamma \subset \partial \Omega$ and at any point *x* of $\Gamma$, we have $N(x)\wedge X(x)=0$ (where *N* denotes the outer normal vector of $\Omega$, here we are using the fact that the differential of the Tubular Neighborhood map is an isometry on $\Gamma$).

Let $\Lambda$ be a smooth, relatively open subset of $\overline{D^2}$. Let *f* be a smooth function on ${\mathbb {C}}^2$ supported away from $u(\partial \Lambda \cap D^2)$ and such that $I\nabla f(x)\cdot N(x)=0$ for any *x* in a neighborhood of $u(\overline{\Lambda }\cap \partial D^2)$ in $\partial \Omega$. We compute as in (24)

$$\begin{aligned} \int _\Lambda \langle du; d(I(\nabla f)\circ u)\rangle =&\int _{\partial \Lambda }\langle \partial _\nu u, I(\nabla f)\circ u\rangle -\int _{\Lambda }i\overline{g}dg\cdot d(f\circ u)\\ \nonumber =&\int _{\overline{\Lambda }\cap \partial D^2} \langle \partial _\nu u, I(\nabla f)\circ u\rangle -\int _{\overline{\Lambda }\cap \partial D^2}G\, \partial _\tau (f\circ u)\\ \nonumber =&\int _{\overline{\Lambda } \cap \partial D^2}\langle \overline{g}J\partial _\tau u+GI\partial _\tau u, I(\nabla f)\circ u\rangle \\ \nonumber =&\int _{\overline{\Lambda } \cap \partial D^2}\langle X\circ u, I(\nabla f)\circ u\rangle =0, \end{aligned}$$

where the last equality follows from the fact that $N\wedge X=0$ and $I(\nabla f)\cdot N=0$ on $u(\overline{\Lambda }\cap \partial D^2)$. We conclude that *u* is a conformal, $\Omega$-free boundary Hamiltonian stationary Lagrangian map with the localization property, but its Lagrangian angle $\overline{g}$ is not constant. $\square$

## Footnotes

1 The definition of the localization property is motivated by the concept of target harmonic maps introduced in [9], and seems suitable also to study the problem with lower regularity assumptions (with the property holding for almost any domain, as in [9]).

2 To see this, let $\{\eta _n\}_{n\in {\mathbb {N}}}$ be a family of smooth functions compactly supported in $D^2$ and converging to 1 almost everywhere. For any $n\in {\mathbb {N}}$, (4), which holds in the distributional sense, implies that $\int _{\Lambda }\langle -\Delta u, \eta _nI(\nabla f)\circ u\rangle =\int _\Lambda \langle \overline{g}dg\cdot du, \eta _nI(\nabla f)\circ u\rangle$. Taking the limit as $n\rightarrow \infty$, we obtain the desired identity.

3 Note that computation (24) was performed away from the singular points of *u*. However, the same computation is valid for $u\in W^{2,1}\cap C^1(\overline{D^2})$ even if $\Lambda$ contains some singularities, as can be seen by approximating *u* in $W^{2,1}\cap C^1(\overline{D^2})$ by a sequence of smooth functions.

## References

[1] Bourgain, J., Brezis, H., Mironescu, P.: maps with values into the circle: minimal connections, lifting, and the Ginzburg–Landau equation. Publ. Math. Inst. Hautes Études Sci. **99**, 1–115 (2004)

[2] Da Lio, F., Rivière, T.: Three-term commutator estimates and the regularity of 1/2-harmonic maps into spheres. Anal. PDE **4**(1), 149–190 (2011)

[3] Eliashberg, Y., Gromov, M.: Convex symplectic manifolds. In: Several Complex Variables and Complex Geometry, Part 2 (Santa Cruz, CA, 1989), vol. 52, pp. 135–162. Part 2. Proc. Sympos. Pure Math. Amer. Math. Soc., Providence, RI (1991)

[4] Fraser, A., Schoen, R.: Uniqueness theorems for free boundary minimal disks in space forms. Int. Math. Res. Not. IMRN **17**, 8268–8274 (2015)

[5] Gaia, F., Orriols, G., Rivièere, T.: A variational construction of Hamiltonian stationary surfaces with isolated Schoen–Wolfson conical singularities. Commun. Pure Appl. Math. **77**(12), 4390–4431 (2024)

[6] Li, M., Wang, G., Weng, L.: Lagrangian surfaces with Legendrian boundary. Sci. China Math. **64**(7), 1589–1598 (2021)

[7] Luo, Y., Sun, L.: Rigidity theorems for minimal Lagrangian surfaces with Legendrian capillary boundary. Adv. Math. **393**, 108124, 15 (2021)

[8] Nitsche, J.: Stationary partitioning of convex bodies. Arch. Ration. Mech. Anal. **89**(1), 1–19 (1985)

[9] Rivière, T.: The regularity of conformal target harmonic maps. Calc. Var. Partial. Differ. Equ. **56**(4), 117, 15 (2017)

[10] Ros, A., Souam, R.: On stability of capillary surfaces in a ball. Pac. J. Math. **178**, 2 (1997)

[11] Schoen , R.: Special Lagrangian submanifolds. In: Global theory of minimal surfaces, vol. 2, pp. 655–666. Clay Math. Proc. Amer. Math. Soc., Providence, RI (2005)

[12] Schoen, R., Wolfson, J.: Minimizing area among Lagrangian surfaces: the mapping problem. J. Differ. Geom. **58**(1), 1–86 (2001)

[13] Schoen, R., Wolfson, J.: The volume functional for Lagrangian submanifolds. In: Lectures on partial differential equations, vol. 2, pp. 181–191. New Stud. Adv. Math. Int. Press, Somerville, MA (2003)

[14] Souam, R.: On stability of stationary hypersurfaces for the partitioning problem for balls in space forms. Math. Z. **224**, 195–208 (1997)

